# Identification of potential regulatory long non-coding RNA-associated competing endogenous RNA axes in periplaque regions in multiple sclerosis

**DOI:** 10.3389/fgene.2022.1011350

**Published:** 2022-10-17

**Authors:** Hani Sabaie, Sharareh Khorami Rouz, Ghazal Kouchakali, Samaneh Heydarzadeh, Mohammad Reza Asadi, Mirmohsen Sharifi-Bonab, Bashdar Mahmud Hussen, Mohammad Taheri, Seyed Abdulmajid Ayatollahi, Maryam Rezazadeh

**Affiliations:** ^1^ Clinical Research Development Unit of Tabriz Valiasr Hospital, Tabriz University of Medical Sciences, Tabriz, Iran; ^2^ School of Life Sciences, Manipal University, Dubai, United Arab Emirates; ^3^ Department of Medical Genetics, Faculty of Medicine, Tabriz University of Medical Sciences, Tabriz, Iran; ^4^ Department of Pharmacognosy, College of Pharmacy, Hawler Medical University, Erbil, Iraq; ^5^ Center of Research and Strategic Studies, Lebanese French University, Erbil, Iraq; ^6^ Urology and Nephrology Research Center, Shahid Beheshti University of Medical Sciences, Tehran, Iran; ^7^ Institute of Human Genetics, Jena University Hospital, Jena, Germany; ^8^ Phytochemistry Research Center, Shahid Beheshti University of Medical Sciences, Tehran, Iran

**Keywords:** bioinformatic analysis, competing endogenous RNA, long non-coding RNA, microarray analysis, multiple sclerosis, periplaque

## Abstract

Slow-burning inflammation at the lesion rim is connected to the expansion of chronic multiple sclerosis (MS) lesions. However, the underlying processes causing expansion are not clearly realized. In this context, the current study used a bioinformatics approach to identify the expression profiles and related lncRNA-associated ceRNA regulatory axes in the periplaque region in MS patients. Expression data (GSE52139) from periplaque regions in the secondary progressive MS spinal cord and controls were downloaded from the Gene Expression Omnibus database (GEO), which has details on mRNAs and lncRNAs. Using the R software’s limma package, the differentially expressed lncRNAs (DElncRNAs) and mRNAs (DEmRNAs) were found. The RNA interactions were also found using the DIANA-LncBase, miRTarBase, and HMDD databases. The Pearson correlation coefficient was used to determine whether there were any positive correlations between DEmRNAs and DElncRNAs in the ceRNA network. Finally, lncRNA-associated ceRNA axes were created based on co-expression and connections between DElncRNA, miRNA, and DEmRNA. We used the Enrichr tool to enrich the biological process, molecular function, and pathways for DEmRNAs and DElncRNAs. A network of DEmRNAs’ protein-protein interactions was developed, and the top five hub genes were found using Cytoscape and STRING. The current study indicates that 15 DEmRNAs, including *FOS*, *GJA1*, *NTRK2*, *CTNND1*, and *SP3*, are connected to the MS ceRNA network. Additionally, four DElncRNAs (such as *TUG1*, *ASB16-AS1*, and *LINC01094*) that regulated the aforementioned mRNAs by sponging 14 MS-related miRNAs (e.g., *hsa-miR-145-5p*, *hsa-miR-200a-3p*, *hsa-miR-20a-5p*, *hsa-miR-22-3p*, *hsa-miR-23a-3p*, *hsa-miR-27a-3p*, *hsa-miR-29b-3p*, *hsa-miR-29c-3p*, *hsa-miR-34a-5p*) were found. In addition, the analysis of pathway enrichment revealed that DEmRNAs were enriched in the pathways for the “MAPK signaling pathway”, “Kaposi sarcoma-associated herpesvirus infection”, “Human immunodeficiency virus one infection”, “Lipid and atherosclerosis”, and “Amphetamine addiction”. Even though the function of these ceRNA axes needs to be investigated further, this study provides research targets for studying ceRNA-mediated molecular mechanisms related to periplaque demyelination in MS.

## Introduction

Multiple sclerosis (MS) is a neurodegenerative, inflammatory disease affecting the central nervous system (CNS). In MS, it is now understood that axonal loss is the primary cause of permanent neurological impairment. Axonal dissection followed by axonal degeneration are thought to be primarily caused by acute inflammatory demyelination, which generally appears as new white matter lesions. Recent research suggests that slow-burning inflammatory demyelination at the edge of some chronic lesions is a key contributor to disease progression, including progressive axonal loss as well as worsening impairment (Prineas et al., 2001; Frischer et al., 2015; Dal-Bianco et al., 2017; Klistorner et al., 2021). These lesions, commonly referred to as smoldering or chronically active lesions (van der Valk and De Groot, 2000), are distinguished by a fully demyelinated, inactive hypocellular center and low-grade active demyelination in periplaque white matter, which has activated microglia/macrophages with myelin breakdown products (Lieury et al., 2014; Frischer et al., 2015). Despite the fact that the pathogenesis of chronic smoldering lesions is widely understood (Prineas et al., 2001), the periplaque region has received little attention up to this point. Although it is generally acknowledged that a combination of genetic and environmental factors may influence a person’s predisposition for MS, the underlying pathophysiological mechanisms and the sequence of events that contribute to lesion expansion remain poorly understood. The major histocompatibility complex (MHC) predominately determines disease occurrence in the genetically susceptible population, whereas modifiable environmental factors like smoking, Epstein-Barr virus (EBV) infection, increased body mass index (BMI) during adolescence, and low vitamin D levels may influence whether a person develops MS (Amato et al., 2018). The human leukocyte antigen (HLA) locus, which is known to encode molecules involved in essential immune functions, is the first genetic risk factor that was found decades ago (Wang et al., 2022). New research suggests that non-coding RNAs (ncRNAs), particularly long non-coding RNAs (lncRNAs) and microRNAs (miRNAs), have the potential to regulate gene expression and offer novel insights into the development of MS (Sheng et al., 2015; Yang et al., 2018; Ghafouri-Fard and Taheri, 2020).

The competing endogenous RNA (ceRNA) hypothesis states that ncRNAs, which serve as miRNA “sponges,” compete with target mRNAs for binding to miRNAs as a novel regulatory mechanism (Salmena et al., 2011). This theory proposes that cross-talk between RNAs, including coding RNAs and ncRNAs, through miRNA complementary sequences known as miRNA response elements (MREs) generates a large-scale regulatory network throughout the transcriptome. If two RNA transcripts control each other *via* a ceRNA-mediated mechanism, then, according to the ceRNA hypothesis, the expression levels of these two RNA transcripts would be negatively correlated with the levels of target miRNAs and positively correlated with each other (Salmena et al., 2011). A few studies have suggested that MS may be caused by altered ceRNA-mediated gene regulation (Bian et al., 2020; Ding et al., 2021; Hao et al., 2022; Karimi et al., 2022; Wang et al., 2022). For instance, it has been demonstrated that the lncRNA *Gm15575* is aberrantly expressed in MS patients and affects the functionality of Th17 in MS *via* ceRNA patterns (Bian et al., 2020). In a separate research, it was found that *HOTAIR* functions as a sponge for *miR-136-5p*, increasing *AKT2*-mediated NF-kB activation and hence favoring the microglial shift towards a proinflammatory M1-like phenotype (Duan et al., 2018), which is harmful in MS. Furthermore, lncRNA *GAS5* has been postulated as a ceRNA for *miR-137* involved in demyelination (Senousy et al., 2020). It is important to do more research on the associated expression patterns and processes in MS due to the fact that the so-called study on the ceRNA axes implicated in MS, particularly in the periplaque region, has received little attention to date. RNAs have greater “druggability” than proteins because their targeting primarily depends on sequence complementarity. Due to their unique properties, these molecules can be easily and inexpensively manufactured into novel targets for RNA-related drugs (Yu et al., 2020). CeRNA interaction networks involve a variety of factors, making it possible to study complex conditions like MS focusing on only one of the potential treatment targets (i.e., an instant variation in the levels of multiple disease-related RNAs) (Moreno-García et al., 2020).

This study aimed to identify the expression profiles and associated lncRNA-associated ceRNA regulatory axes in the periplaque region of MS patients using a bioinformatics approach.

## Methods

In the current study, we used a system biology method for mining expression data related to periplaque regions in the spinal cord of MS with the accession number GSE52139. Our purposes were recognition of the differentially expressed lncRNAs (DElncRNAs) and mRNAs (DEmRNAs) and to develop lncRNA-associated ceRNA regulatory axes by using previous bioinformatics approaches (Sabaie et al., 2021a; Sabaie et al., 2021b).

### Data preparation of the gene expression profile

The gene expression profiles were downloaded from the NCBI Gene Expression Omnibus dataset (GEO, https://www.ncbi.nlm.nih.gov/geo/). The platform of GPL570 (HG-U133_Plus_2) Affymetrix Human Genome U133 and 2.0 Array (chip-based) was used for the mentioned database, in which both information of lncRNA and mRNA were included in the GPL570. The GSE52139 includes sixteen spinal cord samples. Eight periplaque samples were compared to normal-appearing white matter from identical patients (Lieury et al., 2014).

### Data preprocessing and identifying DEmRNAs and DElncRNAs

Background correction and quantile normalization were done through the Robust Multichip Average (RMA) for the total files of data (Irizarry et al., 2003). The AgiMicroRna Bioconductor package version 2.46.0 was used for quality control (Romero and AgiMicroRna, 2020). The differential expression gene analysis (DEGA) was done by the limma package of R (version 3.52.2) (Ritchie et al., 2015) in Bioconductor (https://www.bioconductor.org/) (Huber et al., 2015). The linear model was accomplished *via* limma’s lmFit () function doing on the main factor “group” (i.e., periplaque against normal-appearing white matter). Subsequently, limma’s eBayes () function was applied to calculate differentially expressed genes from the linear fit model. Next, the whole list of lncRNAs’ genes was obtained (https://www.genen ames. org/) through approved HUGO Gene Nomenclature Committee (HGNC) symbols (Braschi et al., 2019). The dataset’s gene symbols were compared with the list of lncRNA genes, and the overlapped genes were chosen. According to this approach, we captured 1,070 lncRNAs. The paired student’s t-test was used to identify statistically significant mRNAs and lncRNAs. The following cut-offs were established for aberrantly expressed genes: (Prineas et al., 2001): |log2 fold change (log2FC) ≥ 1, and *q* < 0.01 (*q* is the False Discovery Rate (FDR) corrected *p*-value) for DEmRNAs and (Dal-Bianco et al., 2017) (log2FC) ≥ 0.585, and *q* < 0.01 for DElncRNAs. The implementation of less stringent selection criteria was prompted by the lower expression level of lncRNAs relative to mRNAs. Finally, using the Pheatmap (version 1.0.12) and the Enhanced Volcano (version1.14.0) R packages, the heat map and the volcano plot of DEGs were depicted.

### RNA interaction pairs prediction

In this study, the DIANA-LncBase v3 (Karagkouni et al., 2020) was utilized to predict the interaction of the lncRNAs and miRNAs, which were experimentally confirmed. The criteria utilized in the DIANA-LncBase query were High “miRNA Confidence Levels” and *Homo* Sapiens “Species”. Additionally, the MS-related miRNAs were collected from the Human microRNA Disease Database (HMDD) version 3.2 (Huang et al., 2019). Also, interactions between the target mRNAs and collected miRNAs (used the MS-related miRNAs) were attained from miRTarBase (including persistent experimental shreds of evidence) (Huang et al., 2020). Then, these newly attained mRNAs were compared to previous mRNAs attained, and consequently, the duplicate mRNAs were used to construct the lncRNA-miRNA-mRNA ceRNA axes.

### Analysis of correlation between DEmRNAs and DElncRNAs, and lncRNA-associated ceRNA axes construction

The Pearson correlation analysis was done to study the positive correlations in the regulatory axes of ceRNA among DEmRNAs and DElncRNAs. In the reverse expression pattern among DElncRNAs and the targeted DEmRNAs, interacted miRNAs targeted DEmRNAs, and DElncRNAs were removed from the network of ceRNA. For correlations’ visualization and calculation, both packages of Hmisc (version 4.7-1) and corrplot (version 0.92) were used. The Pearson correlation coefficients above 0.5 and *p* < 0.05 were considered inclusion criteria. Finally, for the construction of ceRNA regulatory axes, we applied the Cytoscape software (version 3.8.0) (Shannon et al., 2003).

### DEmRNAs and DElncRNAs enrichment analysis

Biological process (BP), molecular function (MF) and the Kyoto Encyclopedia of Genes and Genomes (KEGG) pathway enrichment analysis of the DEmRNAs in the ceRNA network were obtained *via* the Enrichr tool (Chen et al., 2013; Kuleshov et al., 2016). In addition, BP and MF analyses were performed utilizing FuncPred (https://www.funcpred.com) (Perron et al., 2017) to get deeper insight into the DElncRNAs in ceRNA network.

### Construction of protein-protein interaction network and hub genes identification

A PPI network was made by the online STRING database (https://string-db.org/) (Szklarczyk et al., 2019) to predict the interactive relevance of DEmRNAs encoding proteins. The Cytoscape software was used to visualize the PPI network and the hub genes analysis. Moreover, non-interacting genes were removed from the PPI network to simplify it. Subsequently, the top five genes of the PPI network were evaluated by Maximal Clique Centrality (MCC) method by use of CytoHubba (version 0.1) in Cytoscape (Chin et al., 2014). Compared to the other network scoring methods, MCC performed better (Chin et al., 2014).

### Restoration of the subceRNA network

The DElncRNAs and miRNAs relevant to the primary structure of ceRNA were extracted and utilized for the network restoration of the ceRNA network hub genes.

## Results

### DEmRNAs and DElncRNAs identification

At first, background adjustment, normalization, and batch adjustment were performed. Following the normalization, box plots were illustrated to analyze the data distribution in the gene expression data (Appendix I). The correct adjustment was approved by the outcomes from distinct box plot arrays that show similar medians for expression levels.

**FIGURE 1:**
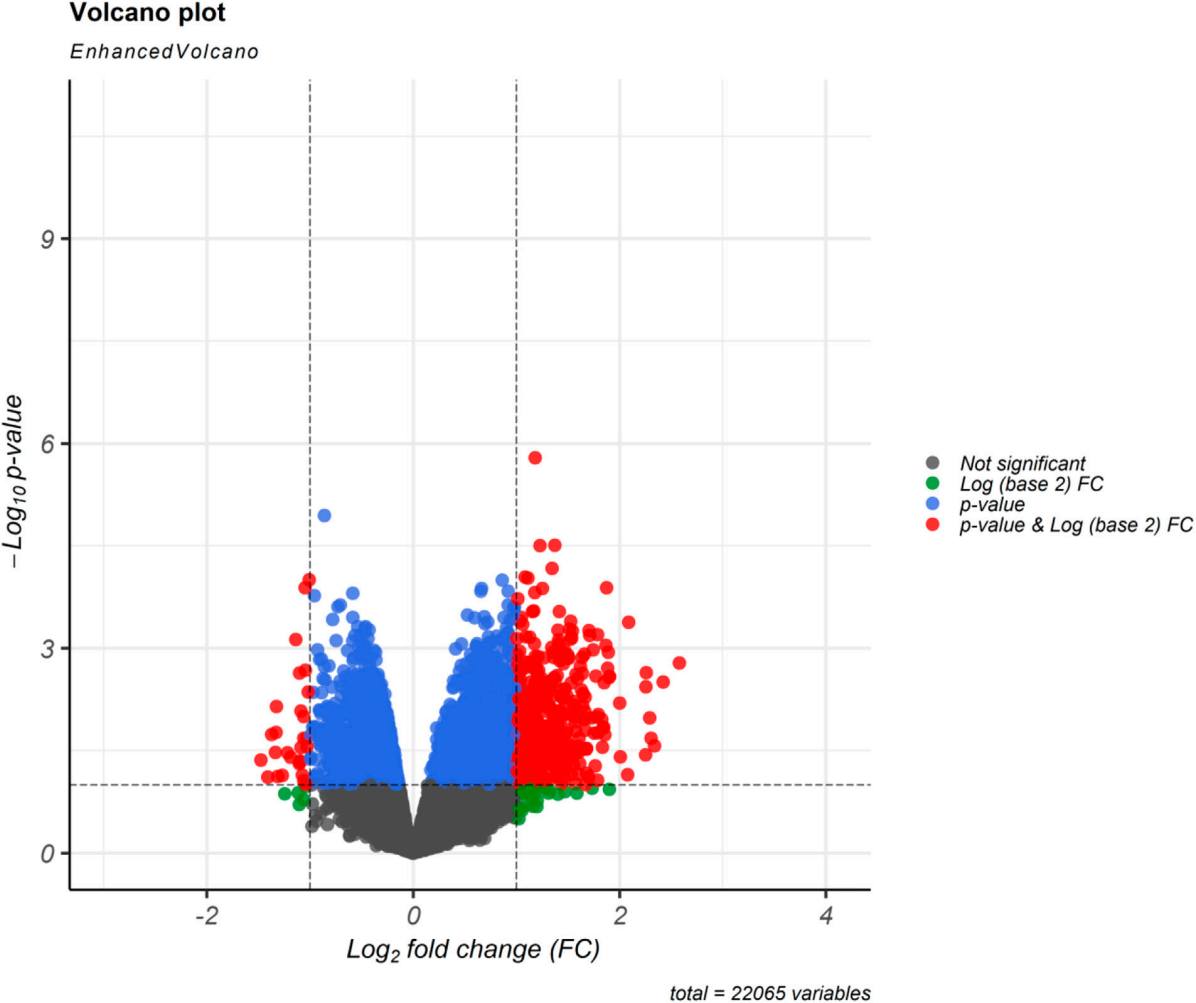
Differentially expressed mRNAs (DEmRNAs) volcano plot. A |(log2FC)| ≥ 1 and an *p* < 0.01 were utilized for screening DEmRNAs.

**FIGURE 2:**
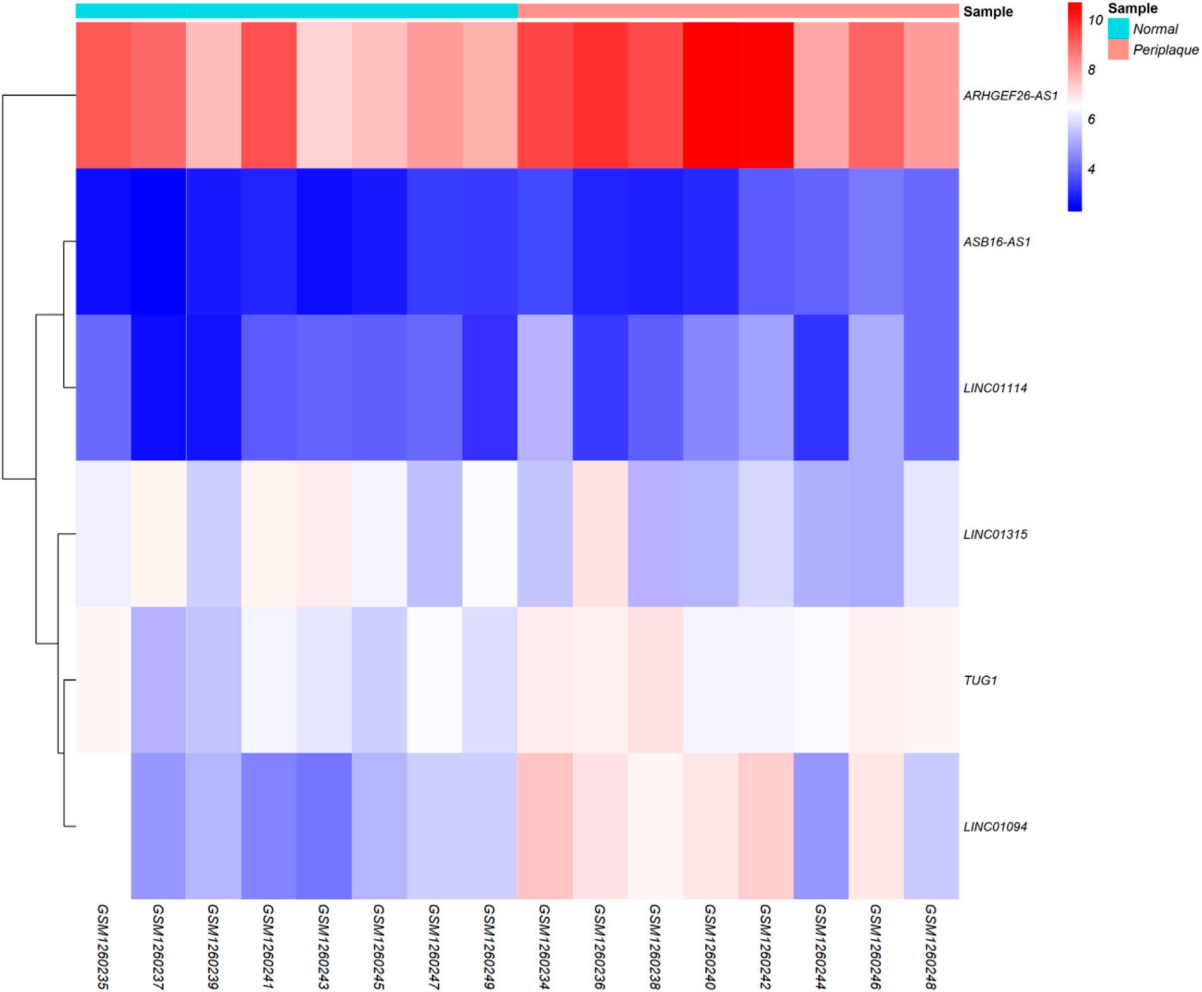
Differentially expressed long non-coding RNAs (DElncRNAs) Heatmap. Lowly expressed DElncRNAs are displayed in blue, while those that are highly expressed are displayed in red.

Regarding to the chosen criteria (|log2FC | ≥ 1, and *q* < 0.01 for DEmRNAs and |log2FC | ≥ 0.585, and *q* < 0.01 for DElncRNAs), we detected 193 DEmRNAs and seven DElncRNAs between periplaque and normal-appearing white matter samples. The DEmRNAs volcano plot and DElncRNAs heat map are shown in Figures 1, 2, respectively. Furthermore, details of DEGs are summarized in Appendix II and III.

### Predicting RNA interaction pairs

Six of the seven DElncRNAs might be targeted *via* the candidate miRNAs. Then, the interplay between candidate miRNAs (obtained from HMDD) and mRNAs was uncovered by miRTarBase. Eventually, 19 overlapping genes were obtained after comparing the candidate mRNAs and DEmRNAs.

### Analysis of relationship between DElncRNAs and DEmRNAs, and lncRNA-associated ceRNA axes construction

**FIGURE 3:**
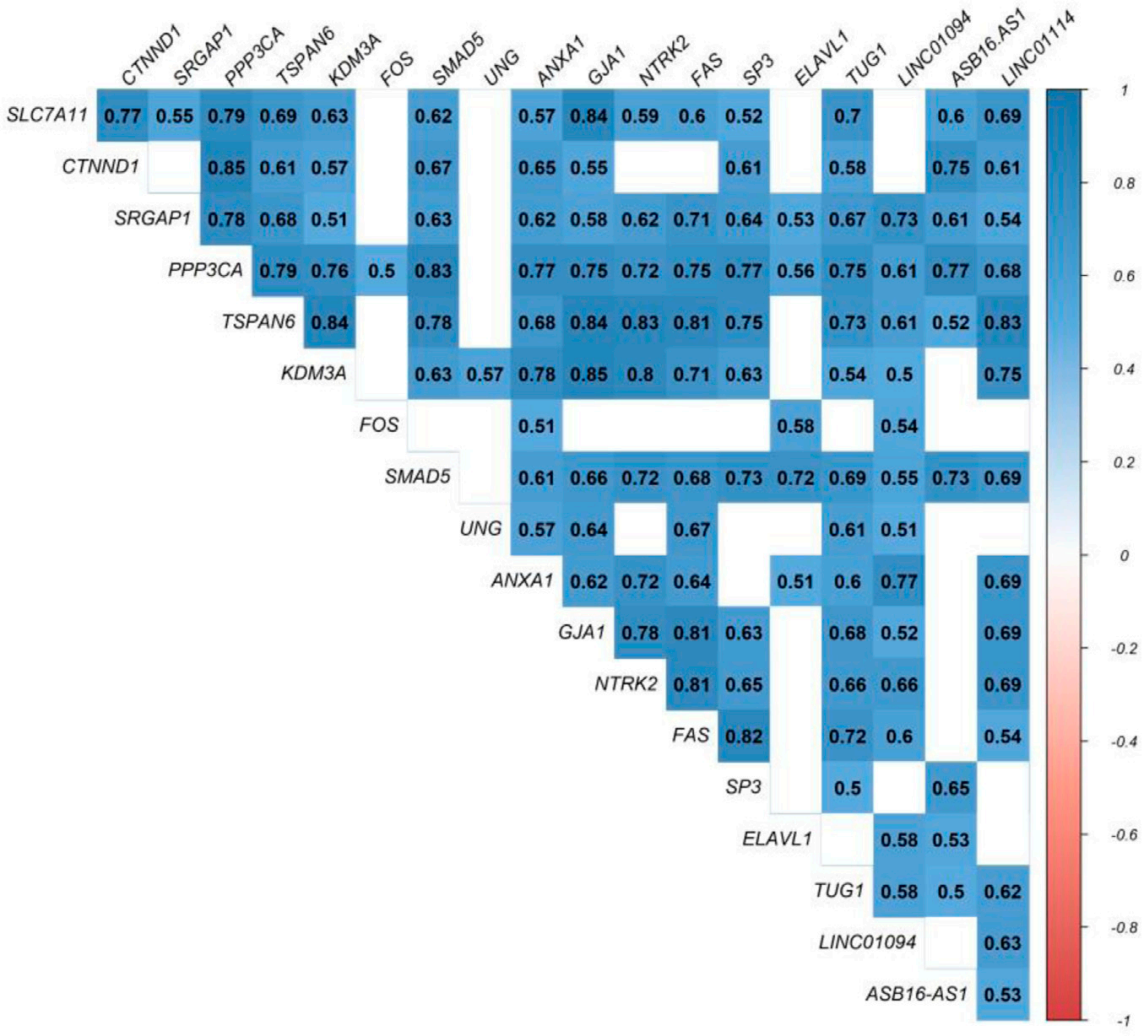
Pearson correlation analysis. Blue is used to represent positive correlations, while red is used to represent negative correlations. *p* greater than 0.05 are regarded as being insignificant (blank), and the intensity is related to correlation coefficients.

**FIGURE 4:**
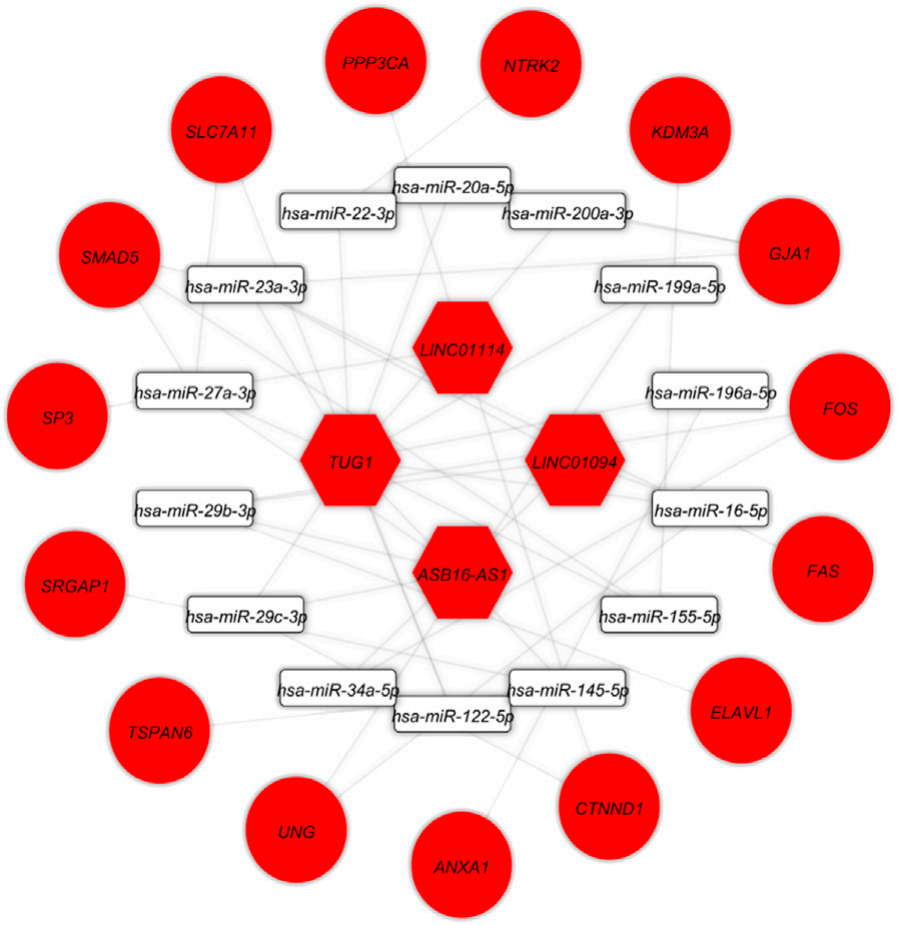
Long non-coding RNA (lncRNA)-associated competing endogenous RNA axes. Red represents upregulation of ceRNAs. LncRNAs, miRNAs, and mRNAs are represented by hexagon, round rectangle, and ellipse, respectively.

At this level, to prove the assumption of ceRNA axes, i.e., lncRNA positively regulates (*via* miRNA interaction) mRNA’s expression; hence, we applied the Pearson correlation analysis among DEmRNAs and DElncRNAs (Figure 3). According to the interactions between DElncRNA-miRNA-DEmRNA and co-expression associations, we constructed the ceRNA regulatory axes to declare the related ceRNA axes with lncRNAs in periplaque regions in MS (Figure 4). Table 1 elucidates the ceRNA axes characteristics, including four DElncRNAs, 14 miRNAs, and 15 DEmRNAs, in total.

**TABLE 1:** Details of competing endogenous RNA axes.

DElncRNA(s)	Shared miRNA	DEmRNA(s)	Expression of DElncRNA(s) and DEmRNA(s)
TUG1	hsa-miR-122-5p	SLC7A11	Upregulation
TUG1	hsa-miR-145-5p	CTNND1	Upregulation
		PPP3CA	
		SRGAP1	
		TSPAN6	
TUG1	hsa-miR-155-5p	KDM3A	Upregulation
		SMAD5	
LINC01094	hsa-miR-16-5p	UNG	Upregulation
TUG1			
TUG1	hsa-miR-196a-5p	ANXA1	Upregulation
TUG1	hsa-miR-199a-5p	UNG	Upregulation
TUG1	hsa-miR-200a-3p	GJA1	Upregulation
TUG1	hsa-miR-20a-5p	GJA1	Upregulation
TUG1	hsa-miR-22-3p	NTRK2	Upregulation
LINC01094	hsa-miR-23a-3p	FAS	Upregulation
TUG1		GJA1	
		SMAD5	
TUG1	hsa-miR-27a-3p	SLC7A11	Upregulation
ASB16-AS1		SMAD5	
LINC01114			
TUG1			
AB16-AS1	hsa-miR-27a-3p	SP3	Upregulation
ASB16-AS1	hsa-miR-29b-3p	ELAVL1	Upregulation
LINC01094			
LINC01094	hsa-miR-29b-3p	FOS	Upregulation
ASB16-AS1	hsa-miR-29c-3p	CTNND1	Upregulation
TUG1			
LINC01094	hsa-miR-34a-5p	FOS	Upregulation

### DEmRNAs and DElncRNAs enrichment analysis

**FIGURE 5:**
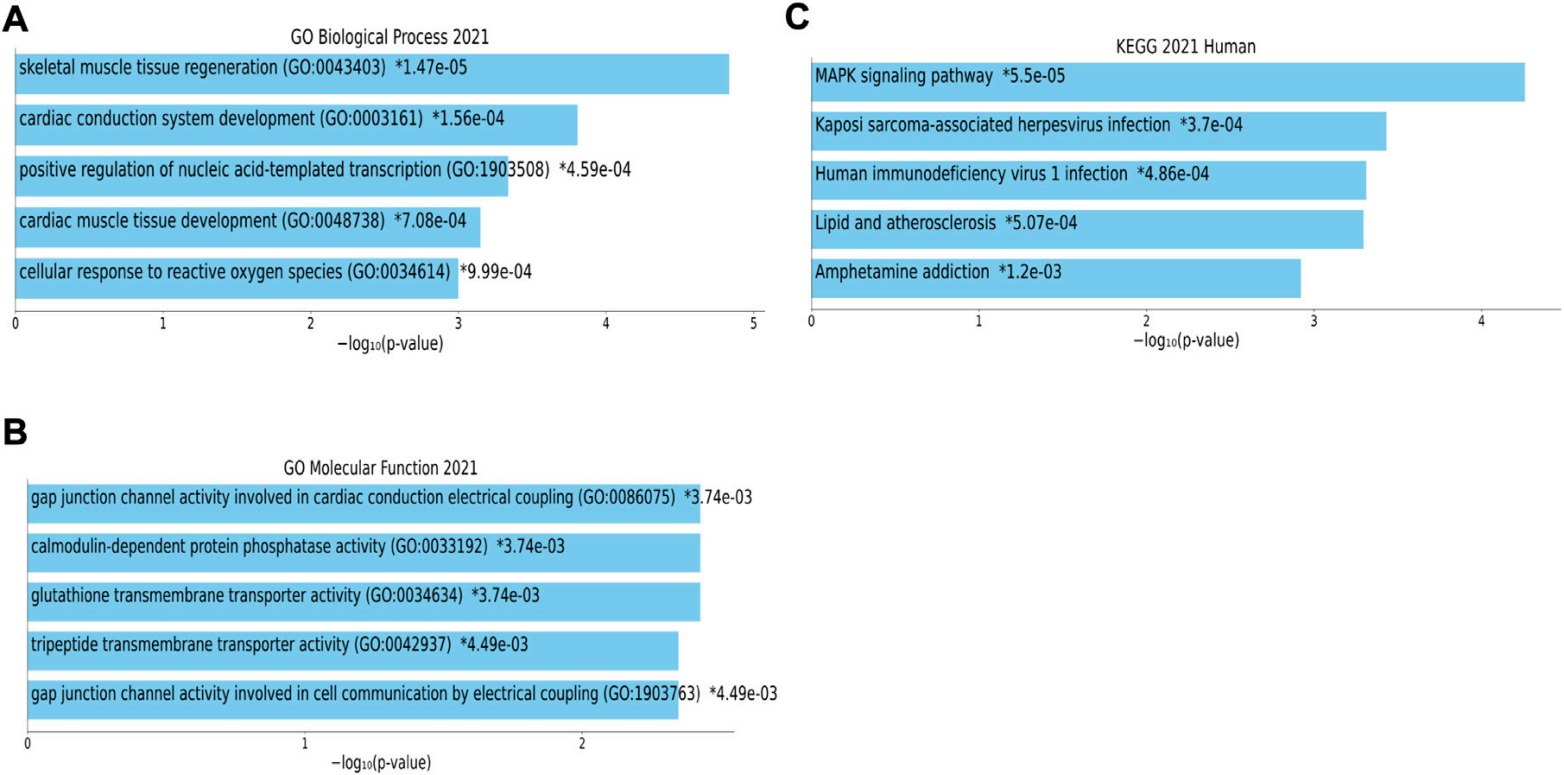
Biological process (BP), molecular function (MF) and Kyoto Encyclopedia of Genes and Genomes (KEGG) pathway enrichment analysis. The top five enriched terms of **(A)** BP, **(B)** MF, and **(C)** KEGG are displayed along with the corresponding *p*-values in a bar graph. Those with significant *p*-values (i.e., <0.05) are matched with the colored bars. A *p*-value (shown with *) shows the significant adjusted *p*-value (<0.05).

Functional annotation obtained on whole DEmRNAs in the ceRNA network. Figures 5A,B indicated that target mRNAs were associated with “skeletal muscle tissue regeneration”, “cardiac conduction system development”, “positive regulation of nucleic acid-templated transcription”, “cardiac muscle tissue development”, “cellular response to reactive oxygen species”, “gap junction channel activity involved in cardiac conduction electrical coupling”, “calmodulin-dependent protein phosphatase activity”, “glutathione transmembrane transporter activity”, “tripeptide transmembrane transporter activity”, and “gap junction channel activity involved in cell communication by electrical coupling”. Moreover, the top enriched KEGG pathways were as follows: “MAPK signaling pathway”, “Kaposi sarcoma-associated herpes virus infection”, “Human immunodeficiency virus one infection”, “Lipid and atherosclerosis”, and “Amphetamine addiction” (Figure 5). The complete list of the enrichment analysis of DElncRNAs is included in Supplementary Files S1–S4.

### Construction of PPI network and identification of hub genes

**FIGURE 6:**
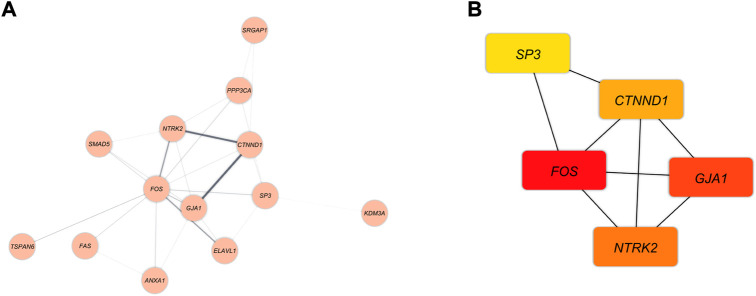
**(A)** The DEmRNAs interactions are shown as blue nodes in this PPI network. **(B)** The recognized hub genes in the PPI network. The yellow, orange, and red nodes, respectively, represent hub genes with weak, moderate, and strong connections.

The PPI network was demonstrated in Figure 6A. The MCC method was applied to assess the top five genes in the PPI network (Figure 6B). The Hub genes with the strongest association are shown with nodes in red color. Besides, orange and yellow nodes illustrate the hub genes with moderate and poor connections, respectively. Fos Proto-Oncogene (*FOS*) and Gap Junction Protein Alpha 1 (*GJA1*) had the strongest correlation. The Neurotrophic Receptor Tyrosine Kinase 2 (*NTRK2*) and Catenin Delta 1 (*CTNND1*) had moderate relevance. In contrast, Sp3 Transcription Factor (*SP3*) demonstrated poor connection.

### Reconstruction of the subceRNA network

**FIGURE 7:**
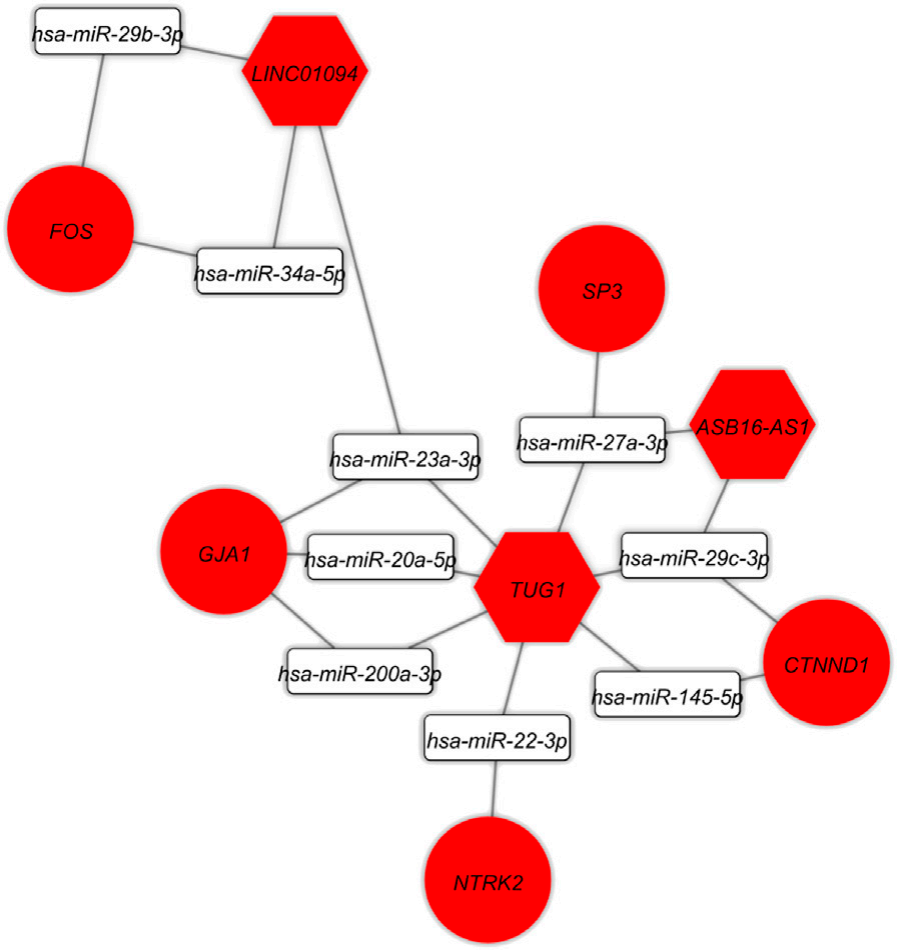
The lncRNA-miRNA-hub gene subceRNA axes. Red represents upregulation of ceRNAs. LncRNAs, miRNAs, and mRNAs are represented by hexagon, round rectangle, and ellipse, respectively.

The DElncRNA-DEmiRNA-hub genes sub-network was reconstructed by use of the hub genes. There were three DElncRNAs (*TUG1*: Taurine upregulated 1*, ASB16-AS1*: ASB16 Antisense RNA 1*,* and *LINC01094*: Long Intergenic Non-Protein Coding RNA 1094), nine miRNAs (*hsa-miR-145-5p*, *hsa-miR-200a-3p, hsa-miR-20a-5p*, *hsa-miR-22-3p*, *hsa-miR-23a-3p*, *hsa-miR-27a-3p*, *hsa-miR-29b-3p*, *hsa-miR-29c-3p*, *hsa-miR-34a-5p*) and five hub genes (*FOS, GJA1, NTRK2, CTNND1, SP3*) overall (Figure 7).

## Discussion

Several studies have found that ceRNA regulatory axes and connected networks play an active role in a variety of developmental and pathological processes, including tumor development and a variety of brain-related illnesses (Ala, 2020; Moreno-García et al., 2020). On the basis of tissue-specific, cellular, and subcellular circumstances, the ceRNA expresses itself differently. In addition to mRNAs, a network may contain a variety of ceRNAs, such as lncRNAs, circRNAs, and pseudogenes. One of the primary RNA types, lncRNAs, is found in the ceRNA machinery and exerts a major impact on biological pathways in both healthy and diseased states (Cai and Wan, 2018). Currently, there is complete consensus that lncRNA expression varies depending on the tissue, cellular type, and developmental stage. In addition to subcellular dispersions, such a distinct tissue dependence is a clear sign that the expression of lncRNAs is tightly regulated (Gloss and Dinger, 2016). The theoretic ideas indicate that the ceRNA regulatory axes linked to lncRNAs can significantly contribute to MS pathogenicity. Based on these ceRNA axes and connections between DElncRNA and DEmRNA, we found the DElncRNA-miRNA-DEmRNA network in the current study. This network contained three important DElncRNAs (*TUG1*, *ASB16-AS1*, and *LINC01094*), nine important miRNAs (*hsa-miR-145-5p*, *hsa-miR-200a-3p*, *hsa-miR-20a-5p*, *hsa-miR-22-3p*, *hsa-miR-23a-3p*, *hsa-miR-27a-3p*, *hsa-miR-29b-3p*, *hsa-miR-29c-3p*, *hsa-miR-34a-5p*) and five hub genes (*FOS*, *GJA1*, *NTRK2*, *CTNND1*, *SP3*).

*TUG1* is a 7.1-kb lncRNA whose function is connected to the normal development of the retina and nervous system. It was first found to be upregulated in response to taurine treatment of developing retinal cells (Young et al., 2005). In experimental autoimmune encephalomyelitis (EAE) mice and lipopolysaccharide-induced BV2 cells, Yue and colleagues discovered that down-regulation of *TUG1* attenuates MS through the inhibition of inflammation by sponging *miR-9-5p via* targeting NF-κB1/p50 (Yue et al., 2019). Multiple lines of research have shown that patients with secondary progressive (Santoro et al., 2020) and relapsing-remitting (Santoro et al., 2016; Dastmalchi et al., 2018) MS exhibit increased *TUG1* expression. For instance, reverse transcription quantitative PCR (RT-qPCR) analysis showed that *TUG1* expression was significantly higher in peripheral serum (Santoro et al., 2016; Santoro et al., 2020) and whole blood (Dastmalchi et al., 2018) in patients with MS compared with controls. These results support our findings that *TUG1* plays a role in the pathophysiology of MS. *TUG1* was, however, downregulated in peripheral blood mononuclear cells (PBMCs) from relapsing-remitting MS patients, according to another expression study (Fenoglio et al., 2018). According to the authors, this contradictory outcome may be due to the various biological sources used for the analysis or a potentially active role for *TUG1* in intercellular communication. Its increased levels in the bloodstream could result from exosomes, an enriched source of ncRNA, releasing it into the cellular environment (Fenoglio et al., 2018). *ASB16-AS1* is localized to 17q21 and is approximately 2,275 bp (Li et al., 2021). The lncRNA *ASB16-AS1* regulates cell proliferation, migration, invasion, and apoptosis through a ceRNA manner in a number of cancers, including glioma (Zhang et al., 2019) and cervical cancer, according to earlier research (Liu et al., 2020). A new long intergenic nonprotein coding RNA called *LINC01094* is found on chromosome four and functions as a ceRNA in a variety of conditions, such as calcified aortic valve disease (Huang et al., 2022), clear cell renal cell carcinoma (Jiang et al., 2020), schizophrenia (Sabaie et al., 2021c), and glioma (Liu et al., 2022). Since our study is the first to identify a link between *ASB16-AS1*, *LINC01094*, and MS, the findings should be confirmed through further research. In agreement with our findings, glioma (Zhang et al., 2019) and schizophrenia (Sabaie et al., 2021c), two additional nervous system diseases, exhibit significantly higher expression levels of *ASB16-AS1* and *LINC01094*, respectively.

We predicted that the sponging of nine key miRNAs (*hsa-miR-145-5p*, *hsa-miR-200a-3p*, *hsa-miR-20a-5p*, *hsa-miR-22-3p*, *hsa-miR-23a-3p*, *hsa-miR-27a-3p*, *hsa-miR-29b-3p*, *hsa-miR-29c-3p*, *hsa-miR-34a-5p*) by key lncRNAs might influence target genes. MiRNAs regulate target gene expression by binding to the non-transcript site of the targeted gene, thereby influencing signal transduction and biological pathways within a cell, which may have an impact on the beginning and progression of MS (Caputo et al., 2015). Based on the HMDD database, the majority of the mentioned miRNAs fall under the circulation biomarker class of literature evidence. However, their precise roles in MS are still unknown. Although our findings are supported by the dysregulation of these miRNAs in MS, the predicted ceRNA axes still need to be verified using molecular methods.

The enrichment analysis results indicated that DEmRNAs’ functions were focused on different BP and MF, including skeletal muscle characteristics, cardiac function, gap junction, protein phosphatase activity, and transmembrane transporter activity. Interestingly, the majority of the DEmRNAs in our study were found to be involved in the “MAPK signaling pathway”, “Kaposi sarcoma-associated herpesvirus infection”, “Human immunodeficiency virus one infection”, “Lipid and atherosclerosis”, and “Amphetamine addiction”, according to the results of our KEGG enrichment analysis. According to the data, the improper (over)activity of the mitogen-activated protein kinase pathway ERK (MAPK^ERK^) causes microglial dysfunction. These findings concern both biochemistry and epigenetics, and they all point to the involvement of this pathway. Recent preclinical studies on neurodegeneration have already suggested that MAPK pathways, in particular MAPK^ERK^, are involved in the process. This is crucial because it has been discovered that microglia with overactive MAPK disrupt local oligodendrocytes, which can result in locoregional demyelination, a characteristic of MS. This represents a novel idea in the pathophysiology of MS in contrast to the prevalent theory of autoimmunity. Smoking, hypovitaminosis D, and EBV infection are all known risk factors for MS that inhibit the MAPK^ERK^ negative feedback phosphatases that normally control MAPK^ERK^ activity. Due to inappropriate MAPK^ERK^ overactivity and subsequent neurodegeneration, these factors may be involved. A contributing factor in the pathophysiology of MS is MAPK^ERK^ overactivity in microglia, which may also explain why MS patients continue to experience neurodegeneration despite receiving optimal immunosuppressive or immunomodulatory therapy (ten Bosch et al., 2021). MS and Kaposi’s sarcoma, for many years, have been suspected to be of viral origin (Enbom, 2001; Tully et al., 2015; Walker and Brew, 2016). New lymphotropic herpesviruses have recently been linked to both of these diseases. Many potential oncogenes found in the viral genome are thought to have originated from the human genome during evolution. In conclusion, the full range of human diseases caused by these viruses is still not fully understood, and the fast-moving field of molecular biology will continue to provide fresh insights into how herpesviruses interact with their hosts (Enbom, 2001). HIV infection and MS are common and well-researched nosological entities. However, MS and HIV co-morbidity have only occasionally been documented in the medical literature. Recent epidemiological studies showed a decreased risk of MS in HIV-positive patients, indicating a negative association between MS and HIV. Increasing clinical evidence also points to a potential lower MS relapse rate in HIV patients. However, it is currently unknown whether this inverse correlation was caused by HIV infection, HIV treatment, or a combination of the two (Stefanou et al., 2019). When compared to the general population, MS patients were reported to have a higher mortality rate, which was attributed to a number of things, including the population’s higher prevalence of cardiovascular diseases (Manouchehrinia et al., 2016). Strong evidence links MS to an increased risk for stroke, myocardial infarction, and heart failure (Christiansen et al., 2010). The primary causes of vascular dysfunction in MS are not fully understood, but the presence of chronic inflammation and autoimmunity, oxidative stress, and the dysregulation of the cardiovascular autonomic in MS patients have all been proposed as potential causes. Atherosclerosis, arteriosclerosis, and endothelial dysfunction are all significantly influenced by inflammation. Additionally, it permits plaque rupture, which raises the danger of acute coronary syndrome (Crea and Libby, 2017). The amphetamines have central stimulant action and sympathomimetic properties. Clinical signs of toxicity include sudden death owing to psychosis, rhabdomyolysis, cardiac arrhythmia, stroke, and seizures, which are comparable to those of cocaine (Weis et al., 2011). Abuse of amphetamines can lead to neuropathologic changes such as cerebral infarcts and hemorrhages, which are frequently brought on by emboli connected to cardiac arrhythmias or myocardial infarction (Delaney and Estes, 1980). In addition, vasospasm or vasculitis resulting from the pharmacological effect of amphetamines may be contributing causes (Delaney and Estes, 1980; Weis et al., 2011). Weis et al. (Weis et al., 2011) reported a patient with amphetamine abuse-related acute demyelination that had not been previously described. The first report of amphetamine in the afflicted region of the brain strongly supports a link between amphetamine usage and demyelination; nevertheless, the pathogenetic processes of demyelination are yet unknown. In this study, BP and MF enrichment analyses were also performed on DElncRNAs in the ceRNA network. The findings suggested that the majority of DElncRNAs’ activities were focused on neuron recognition, synaptic function, ion channel activity, and transcriptional and post-transcriptional regulation.

We identified five hub genes in the ceRNA network, including *FOS*, *GJA1*, *NTRK2*, *CTNND1*, and *SP3*. FOS is a transcription factor linked to neuronal activity. Leucine zipper proteins from the *FOS* gene family can dimerize with JUN family proteins to form the transcription factor complex JUN (Shang et al., 2020). FOS may have essential regulatory roles in modulating gene expression in the pathophysiology of MS (Liu et al., 2013; Kotelnikova et al., 2015), as well as in the mechanism of action of IFNbeta1a and fingolimod (Howe et al., 2006; Kauffman et al., 2009; Yester et al., 2015; Anastasiadou and Knöll, 2016; Groves et al., 2018; Tran et al., 2020). In agreement with our findings, Yu et al. (1991) discovered a two-fold increase in *FOS* RNA in MS white matter relative to control tissue. The gap junctions (GJs), which form intercellular communication channels between two apposing cells or hemichannels with the extracellular environment, serve essential roles in maintaining small molecule homeostasis (Basu and Sarma, 2018). The GJs of the CNS are essential for maintaining myelin sheath and neuronal function. Connexin (Cx) proteins are the structural components of GJs (Basu and Sarma, 2018). The expression of *GJA1* (*Cx43*) is essential for maintaining K+ buffering and nutritional homeostasis in oligodendrocytes, CNS myelin, and oligodendrocyte function, according to previous research (Basu and Sarma, 2018; Une et al., 2021). Furthermore, ablation of *Cx43* in brain gray matter astroglia reduces the severity of EAE, an animal model of MS, by promoting an anti-inflammatory phenotype in astroglia and suppressing pro-inflammatory activation of spinal microglia partly through decreased cerebrospinal fluid pro-inflammatory cytokine/chemokine levels (Une et al., 2021). Therefore, brain astroglial *Cx43* may be a potential MS therapeutic target (Une et al., 2021). *GJA1* expression was dramatically elevated in the spinal cord periplaques of progressive MS patients and in EAE mice, according to previous research. (Lieury et al., 2014; Jin et al., 2017; Nataf et al., 2017). In addition, Nataf et al. (2017) found an astrocytosis-related co-expression module with the astrocyte gene *GJA1* as its central hub. These findings are consistent with our result, indicating that the *GJA1* gene is involved in the pathophysiology of MS. NTRK2 (also known as TrkB) is a membrane-bound receptor that belongs to the neurotrophic receptor kinase (NTRK) family. When neurotrophic proteins bind, members of the NTRK family and MAPK pathways are phosphorylated, resulting in cell differentiation *via* NTRK2 (Li et al., 2022). The Brain-Derived Neurotrophic Factor (*BDNF*) is one of the most important players in the biological pathways of brain plasticity. BDNF belongs to the neurotrophin family and binds to the NTRK2. The binding causes the MAPK pathway (Chao, 2003) to be activated, regulating synaptic plasticity and repair. On the other hand, fibrinogen (Fg)-containing plaques have been linked to memory loss in inflammatory neurodegenerative diseases such as Alzheimer’s, MS, stroke, and traumatic brain injury (Clark et al., 2018). Data indicate that Fg interacts with astrocytes, resulting in the upregulation of intercellular adhesion molecule 1 (*ICAM-1*) and *TrkB* and the phosphorylation of TrkB, and hence the activation of astrocytes (Clark et al., 2018). Since it is known that activated astrocytes express TrkB, which induces neuronal degeneration (Colombo et al., 2012), the interaction of Fg with astrocytes and the subsequent activation of TrkB may be a process involved in memory loss (Clark et al., 2018). Consistent with our findings, *BDNF* and its receptor truncated *trkB* tyrosine kinase receptor were detected in lesions of MS patients (Stadelmann et al., 2002) as well as EAE (Stadelmann et al., 2002; De Santi et al., 2009; Colombo et al., 2012). The *CTNND1* gene encodes the cellular adhesion protein p120-catenin (p120), which is essential for myelinating Schwann cells, cell-cell contacts, and proper myelin sheath development. (Perrin-Tricaud et al., 2007). By altering Rho GTPase activity, CTNND1 is implicated in the regulation of cadherin-mediated adhesion and dynamic regulation of the actin cytoskeleton (Anastasiadis et al., 2000; Noren et al., 2000; Grosheva et al., 2001). CTNND1 was found among myelin basic protein (MBP) partners by Smirnova et al. (2021). Intrinsically aberrant MBP is one of the most important autoantigens in autoimmune neurodegeneration and MS (Benjamins and Morell, 1978; Garbay et al., 1988). These findings support our findings and show that the *CTNND1* gene contributes to the pathophysiology of MS. Human transcription factor SP3, a member of the Sp1 family that binds GC/T box elements, has the ability to activate or suppress the expression of a number of immune system-related genes (Grekova et al., 2002). Multiple lines of evidence indicate a reduction in *SP3* expression in PBMCs from patients with MS (Grekova et al., 1996; Grekova et al., 2000; Grekova et al., 2002; Lin et al., 2008). This discrepancy between these results and our findings may be explained as follows. The profile of gene expression in PBMCs does not reflect CNS components (Comabella and Martin, 2007; Dutta and Trapp, 2012). In addition, the gene expression profile derived from blood may potentially indicate the influence of variables unrelated to the illness process (Goertsches and Zettl, 2007).

It should be noted that a number of technical factors, including various methodologies, patient characteristics, a sample preparation, data analysis, and platforms, could have an impact on gene expression profiles. Additionally, small sample size may compromise statistical power. Of course, additional experimental studies and comparisons to reanalysis of modified microarray gene expression or other robust methodologies (e.g., WGCNA and ARACNE) are needed to confirm our findings.

## Conclusion

Demyelination of the periplaque is likely to be the first stage of the lesion expansion process, making it a potential target for remyelinating therapies. Our research suggests potential ceRNA-mediated molecular mechanisms for this expansion involving ceRNA. Although the functions of the ceRNA axes need to be studied, this investigation provides potential research targets for examining molecular pathways that may be important for the pathogenesis of MS.

## Data Availability

Publicly available datasets were analyzed in this study. This data can be found here: (https://www.ncbi.nlm.nih.gov/geo/query/acc.cgi?acc=GSE52139).
