# Supplementary material for: Identification of potential regulatory long non-coding RNA-associated competing endogenous RNA axes in periplaque regions in multiple sclerosis
Source: Front Genet. 2022 Oct 17;13:1011350. doi: 10.3389/fgene.2022.1011350 (PMC9619104; doi:10.3389/fgene.2022.1011350)
Supplement: Supplementary file 3 [file Table1.DOCX]

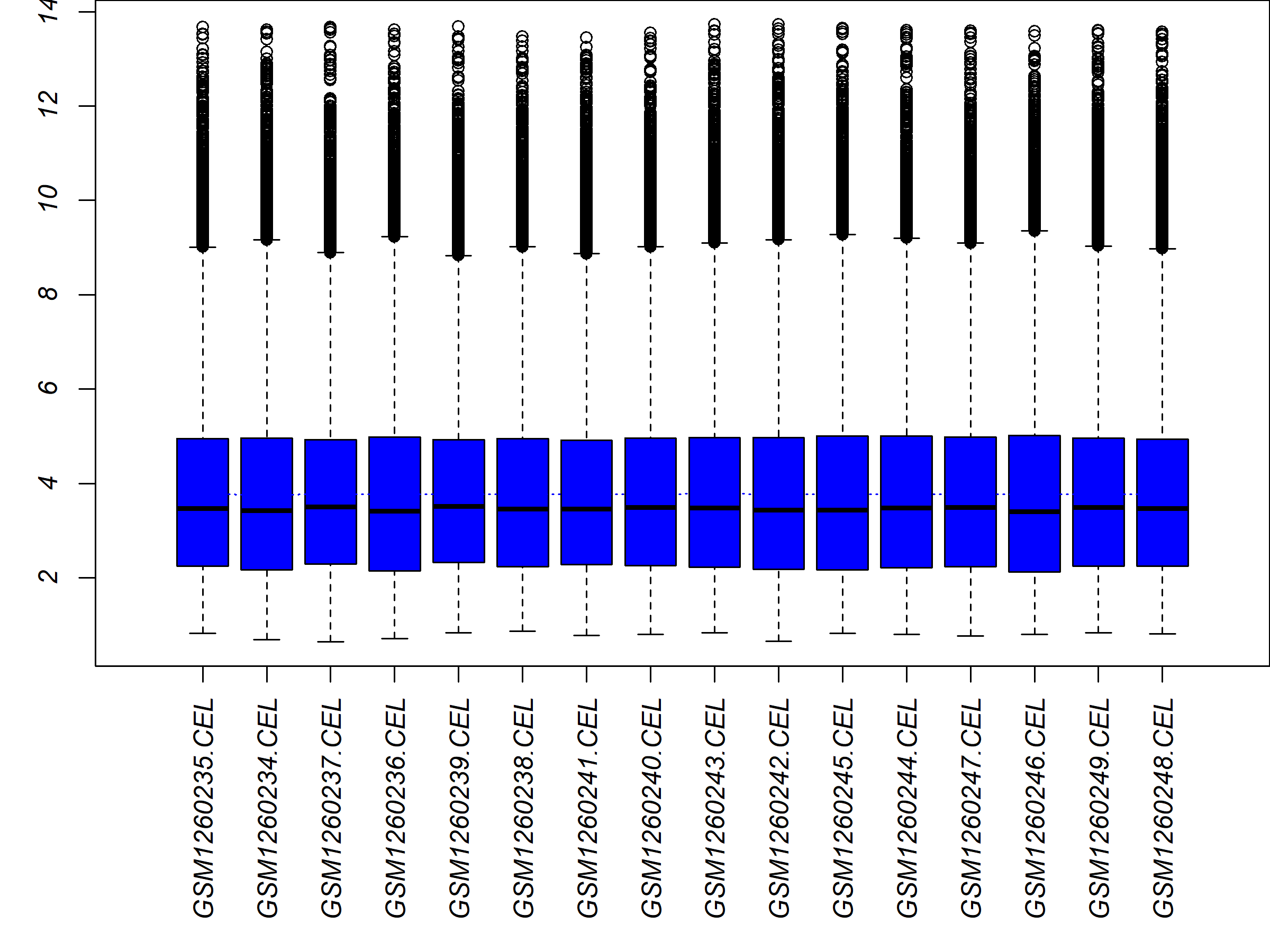


**Figure S1.** Boxplot for GSE52139 dataset. The horizontal axis displays the sample names, while the vertical axis displays the gene expression.
